# Apolipoprotein L2's Role in Liver Fibrosis

**DOI:** 10.1016/j.gastha.2025.100665

**Published:** 2025-03-27

**Authors:** Etienne Pays

**Affiliations:** Laboratory of Molecular Parasitology, Institut de Biologie et de Médecine Moléculaires (IBMM), Université Libre de Bruxelles, Gosselies, Belgium

Apolipoprotein L2 (APOL2) was recently reported to promote liver fibrosis upon transforming growth factor-β1 (TGF-β) stimulation of human hepatic stellate cells.^1^ The use of the potent antiliver fibrosis lead compound 12-deoxyphorbol 13-palmitate (DP) allowed the identification of APOL2 as the specific target. The key involvement of APOL2 in fibrosis was confirmed following experimental ablation of either APOL2 in human hepatic cells or the putative APOL2 ortholog apolipoprotein L8 (APOL8) in mice. Based on structure and activity of the closely related human apolipoprotein L1 (APOL1), together with the high similarity between the APOL1 and APOL2 interactomes, I propose that like APOL1 in kidney podocytes, APOL2 controls Golgi-derived vesicular trafficking in hepatic cells, causing the exocytotic release of fibrogenic extracellular vesicles. Moreover, I provide evidence that the site of DP interaction with APOL2 precisely coincides with an APOL1 cholesterol-binding motif involved in kidney podocyte cytotoxicity causing APOL1 variant nephropathy, linking APOL-induced pathologies of these 2 diseases. I propose that APOL2 trafficking activity requires interaction with membrane lipids of Golgi-derived vesicles.

APOL2 belongs to a protein family containing 6 members in humans and 12 members in mice, expressed at different levels in all organs.^2^ APOL2 is the closest homolog of APOL1 (61.4% identity, 74.6% similarity), and shares the APOL1 N-terminal domain structure.^3^ Both proteins concentrate close to the perinuclear endoplasmic reticulum (ER) and Golgi apparatus,^1^^,^^4^ but APOL1 can also be secreted, owing to the generation of a signal peptide-containing isoform following alternative mRNA splicing. Like APOL1, APOL2 contains a hydrophobic double-stranded hairpin helix potentially able to span a membrane, but not expected to fully insert in the ER or Golgi membrane due to acidic residues hindering transmembrane insertion at neutral pH.^2^ Such strict pH requirement is crucial for intracellular or extracellular APOL1 activities induced by type I-interferon (IFN-I) in kidney podocytes, or for extracellular APOL1 lytic activity on the bloodstream African trypanosome *Trypanosoma brucei brucei*, which allows humans to escape infection by this parasite.^2^ Contrasting with these important similarities, a clear feature distinguishing APOL2 from APOL1, is the fact that unlike APOL1, APOL2 expression is barely affected by IFN-I, but is rather induced by profibrotic stimuli, such as TGF-β.^1^^,^^4^

Different human or murine APOLs are crucially involved in membrane dynamics (traffic, fission, and fusion).^2^^,^^5^^,^^6^ Therefore, such activity could also apply to APOL2. Accordingly, from the proteomic data of APOL2 immunoprecipitates of hepatic cell extracts,^1^ APOL2 appears to share the main APOL1-specific interaction pattern,^4^ characterized by strong association with several actomyosin components involved in membrane dynamics, primarily the heavy and 2 light chains of nonmuscular myosin-2A, namely myosin-heavy chain 9 and myosin-light chains 12B and 6, as well as myosin-heavy chain 10, several tropomyosins and other cytoskeletal proteins. The high level of these interactions cannot be explained by the low coimmunoprecipitation of APOL1.^1^ Thus, APOL2 could be involved in intracellular membrane trafficking.

Considering the extensive similarities of sequence, structure, localization and protein interactions between APOL2 and APOL1, APOL2 may share APOL1-like functions, but under different conditions. In kidney podocytes, APOL1 directs IFN-I–driven trafficking of vesicles carrying the lipid scramblase autophagy-9A from the Golgi to mitochondrion-ER contact sites (MERCS), for induction of mitophagy and apoptosis by APOL1-associated apolipoprotein L3 (APOL3), which controls both fission and fusion of mitochondrial membranes.^2^^,^^4^, ^5^, ^6^ Regarding APOL2, no precise function was identified, except that like APOL1, APOL2 does not directly induce apoptosis.^4^^,^^7^ Thus, in TGF-β–treated liver cells, APOL2 could be involved in vesicular trafficking triggering fibrosis, but not apoptosis. An obvious hypothesis is the role of APOL2 in the release of extracellular vesicles from hepatic stellate cells, which is known to underly liver fibrosis.^8^, ^9^, ^10^ Consistently, several Ras guanosine triphosphate hydrolase–activating proteins and binding proteins, typically linked to vesicular trafficking, are present in APOL2 immunoprecipitates.^1^ I conclude that APOL2-mediated membrane trafficking could induce exocytosis of fibrogenic vesicles.

Upon viral infection, APOL2 is trafficked to MERCS where mitophagy and apoptosis are initiated,^11^ possibly owing to APOL2 association, like APOL1, with the mitophagy receptor prohibitin-2.^1^^,^^5^ During liver fibrosis, activated hepatic stellate cells appear to exhibit inhibition of mitophagy and apoptosis.^12^^,^^13^ Based on information gathered on APOL1 and APOL3, I propose that APOL2 is involved in these inhibitions. In kidney podocytes, mitophagy and apoptosis are strongly inhibited following deletion of the C-terminal region of APOL1 (in APOL1Δ podocytes), due to APOL1 unfolding resulting from the absence of C-terminal leucine zipper helix (LZ2) interaction with a N-terminal LZ helix (LZ1) (Figure A).^4^, ^5^, ^6^ Protein unfolding in APOL1Δ leads to increased accessibility of hydrophobic helices (in orange in Figure A), enhancing their interaction with APOL3, inactivating APOL3 and inhibiting APOL3-induced mitophagy and apoptosis.^4^, ^5^, ^6^ Consistently, the absence of APOL3 (in APOL3 KO podocytes), also results in inhibition of mitophagy and apoptosis.^4^, ^5^, ^6^ Furthermore, the phenotype of APOL1Δ or APOL3 KO podocytes exhibits striking resemblance, albeit more severely, with that of podocytes expressing the west African APOL1 C-terminal variants G1 and G2, which allow human resistance to the APOL1-resistant parasite *Trypanosoma brucei rhodesiense*, but also cause APOL1 variant nephropathy.^4^ Whereas APOL1Δ lacks LZ2, the G1 and G2 variants exhibit LZ2 sequence divergence with APOL1 (Figure B), which also induces their increased interaction with APOL3 and consecutive APOL3 inactivation.^4^ Like in G1 or G2, the LZ2 sequence of APOL2 differs from that of APOL1 (Figure B), and in APOL2, no interaction is observed between LZ2 and LZ1.^2^^,^^4^ Thus, APOL2 folding is expected to differ from that of APOL1 and to resemble that of APOL1Δ (Figure A), and like G1 or G2 in IFN-I–treated podocytes, in TGF-β–treated liver cells APOL2 could inhibit mitophagy and apoptosis through APOL3 inactivation.

**Figure:**
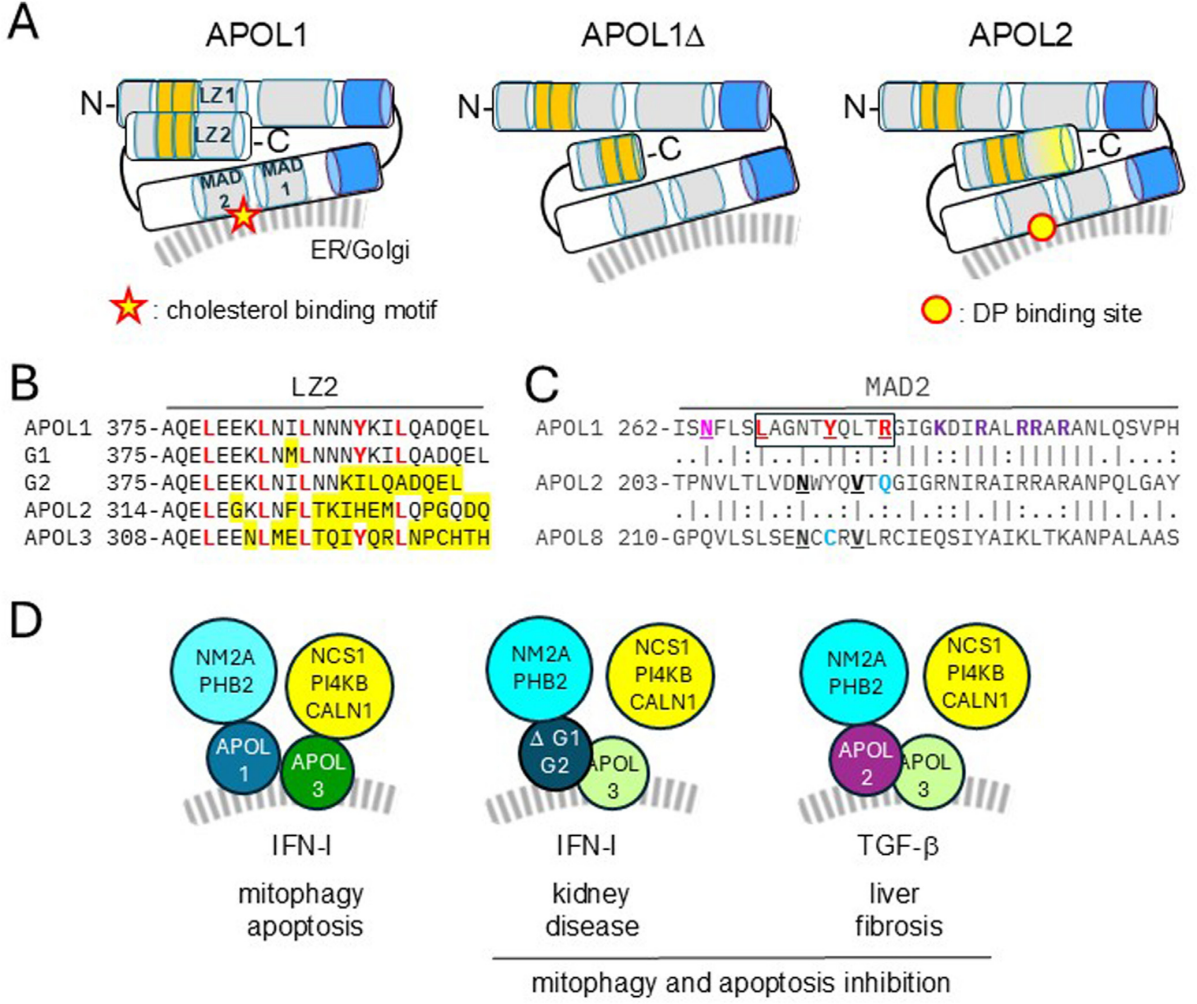
APOL2 structure and activity. (A) Structural difference between APOL2 and APOL1. The protein α-helices are represented as cylinders (hydrophobic helices in orange and potentially transmembrane helices in blue). In APOL1, interaction occurs between N- and C-terminal tandems of hydrophobic and LZ helices. In APOL1Δ, experimentally depleted of LZ2, the lack of LZ2–LZ1 interaction increases the accessibility of hydrophobic helices. In APOL2, LZ2 sequence divergence (yellow) prevents LZ2–LZ1 interaction. (B) LZ2 sequence of different APOLs (divergence with APOL1 in yellow; LZ2 key residues in bold red). In G1, M384 is predicted to destabilize LZ2.^14^ (C) MAD2 sequence of different APOLs. APOL1 MAD2 contains a cholesterol binding motif (boxed; key residues in bold red). N264K mutation prevents G1 or G2 cytotoxicity (N264 in pink). Residues in bold violet may interact with membrane phospholipids. MAD2 of APOL2 or its putative murine ortholog APOL8 contains a DP binding site (key interacting residues in bold black), but no cholesterol-binding motif (disruptive residues in blue). (D) APOL activities. In human podocytes, IFN-I–mediated inflammation triggers Golgi-derived trafficking of vesicles carrying the APOL1/nonmuscular myosin-2A (NM2A)/prohibitin-2 (PHB2) and APOL3/neuronal calcium sensor-1 (NCS1)/PI4-kinase-B (PI4KB)/calneuron-1 (CALN1) complexes, inducing mitophagy and apoptosis. In G1- or G2-expressing individuals, APOL3 is inactivated through increased APOL1 interaction, uncoupling NCS1/PI4KB and CALN1 from APOL3 control. In the liver, TGF-β increases APOL2 expression, leading to fibrosis possibly due to Golgi-derived traffic of vesicles carrying inactivated APOL3. MAD2, membrane-addressing domain 2.

APOL2 was proposed to interact with the sarcoplasmic/ER calcium ATPase-2, triggering Ca^2+^ fluxes inducing ER stress signaling.^1^ However, this signaling could alternatively result from APOL2 vesicular trafficking, like occurs with APOL1 C-terminal variants. Indeed, these variants induce a cascade of pleiotropic podocyte dysfunctions including ER stress and stress-mediated activation of protein kinases, linked to the traffic of vesicles carrying inactivated APOL3.^2^^,^^15^ Such dysfunctions may result from inhibition of the APOL3 control of phophatidylinositol-4-phosphate synthesis. Unlike APOL2, APOL3 directly interacts with PI4-kinase-B (PI4KB), neuronal calcium sensor-1 (NCS1) and calneuron-1 (CALN1), affecting both PI4KB localization and activity at the Golgi.^4^^,^^5^ In particular, APOL3 is required for PI4KB sequestration at the Golgi. I propose that APOL2-mediated inactivation of APOL3, present in APOL2 immunoprecipitates,^1^ allows Golgi-derived trafficking of PI4KB-NCS1 and CALN1 to other cellular membranes. PI4KB-NCS1 and CALN1 can respectively promote vesicle exocytosis at the plasma membrane and sarcoplasmic/ER calcium ATPase-2 activation at the ER,^16^, ^17^, ^18^, ^19^ accounting for both release of extracellular vesicles and increase in ER stress. I conclude that inactivation of APOL3 could be responsible for both ER stress and increased vesicle exocytosis underlying liver fibrosis. In this regard, it would be interesting to investigate if individuals naturally lacking APOL3 following gene mutation,^20^ suffer liver fibrosis even without TGF-β stimulation.

Finally, the antifibrosis DP compound was found to interact with the T203-Y239 region of APOL2, and more particularly with the 2 key residues N212 and V216.^1^ As shown in Figure C, this region corresponds almost exactly to the second helix of the membrane-addressing domain of APOL1. The membrane-addressing domain helices are required for APOL1 interaction with cellular membranes, and appear to be partially buried in the membrane outer layer.^2^^,^^15^^,^^21^ Interestingly, the specific DP targeting to APOL2, but not APOL1,^1^ precisely occurs in a motif responsible for cholesterol binding in APOL1 (Figure C). Interaction of this motif with the podocyte plasma membrane is involved in cytotoxicity associated with APOL1 variant nephropathy, and this cytotoxicity is lost upon N264K mutation, which is expected to prevent cholesterol interaction.^2^^,^^15^ In murine APOL8, considered as the APOL2 ortholog,^1^ the key residues involved in DP binding to APOL2 are conserved. Moreover, in both APOL2 and APOL8, the cholesterol binding motif is disrupted, due to substitution of key residues (Figure C). Because the inactivation of the profibrotic APOL2 activity involves DP targeting to a motif that in APOL1 interacts with cholesterol, I conclude that APOL2 activity depends on interaction with a cholesterol- or DP-like membrane lipid of Golgi or Golgi-derived vesicles.

In summary, upon TGF-β treatment of hepatic cells, APOL2 could mimic the IFN-I–induced APOL1 activity of vesicular trafficking from the Golgi to MERCS, but causing inhibition of mitophagy and apoptosis through APOL3 inactivation like occurs with the C-terminal APOL1 variants APOL1Δ, G1 or G2 (Figure D). APOL3 inactivation would result in ER stress and release of fibrogenic extracellular vesicles, due to delocalization of the APOL3 partners PI4KB-NCS1 and CALN1. It is worth emphasizing that TGF-β induces fibrosis in various organs, like occurs in chronic kidney disease or idiopathic pulmonary fibrosis.^22^, ^23^, ^24^ Whether APOL2 is involved in all cases deserves investigation. Moreover, since IFN-I–induced APOL1 variant nephropathy is linked to both podocyte ER stress and fibrosis,^2^^,^^15^ it is possible that in the kidney, the APOL1 variants G1 and G2 mimic APOL2 activity in the liver.
